# Identification and Characterization of Three Chitinases with Potential in Direct Conversion of Crystalline Chitin into *N*,*N*′-diacetylchitobiose

**DOI:** 10.3390/md20030165

**Published:** 2022-02-24

**Authors:** Xue-Bing Ren, Yan-Ru Dang, Sha-Sha Liu, Ke-Xuan Huang, Qi-Long Qin, Xiu-Lan Chen, Yu-Zhong Zhang, Yan-Jun Wang, Ping-Yi Li

**Affiliations:** 1 State Key Laboratory of Microbial Technology, Institute of Marine Science and Technology, Shandong University, Qingdao 266237, China; 201611626@mail.sdu.edu.cn (X.-B.R.); dangyanru@mail.sdu.edu.cn (Y.-R.D.); 202012644@mail.sdu.edu.cn (S.-S.L.); 202112533@mail.sdu.edu.cn (K.-X.H.); qinqilong@sdu.edu.cn (Q.-L.Q.); cxl0423@sdu.edu.cn (X.-L.C.); zhangyz@sdu.edu.cn (Y.-Z.Z.); 2 College of Marine Life Sciences, Frontiers Science Center for Deep Ocean Multispheres and Earth System, Ocean University of China, Qingdao 266003, China; 3 Laboratory for Marine Biology and Biotechnology, Pilot National Laboratory for Marine Science and Technology, Qingdao 266237, China; 4 Institute of Wetland Agriculture and Ecology, Shandong Academy of Agricultural Sciences, Jinan 250100, China

**Keywords:** chitinases, crystalline chitin, chitooligosaccharides, *N*,*N*′-diacetylchitobiose, *Pseudoalteromonas*

## Abstract

Chitooligosaccharides (COSs) have been widely used in agriculture, medicine, cosmetics, and foods, which are commonly prepared from chitin with chitinases. So far, while most COSs are prepared from colloidal chitin, chitinases used in preparing COSs directly from natural crystalline chitin are less reported. Here, we characterize three chitinases, which were identified from the marine bacterium *Pseudoalteromonas flavipulchra* DSM 14401^T^, with an ability to degrade crystalline chitin into (GlcNAc)_2_ (*N,N*’-diacetylchitobiose). Strain DSM 14401 can degrade the crystalline α-chitin in the medium to provide nutrients for growth. Genome and secretome analyses indicate that this strain secretes six chitinolytic enzymes, among which chitinases Chia4287, Chib0431, and Chib0434 have higher abundance than the others, suggesting their importance in crystalline α-chitin degradation. These three chitinases were heterologously expressed, purified, and characterized. They are all active on crystalline α-chitin, with temperature optima of 45–50 °C and pH optima of 7.0–7.5. They are all stable at 40 °C and in the pH range of 5.0–11.0. Moreover, they all have excellent salt tolerance, retaining more than 92% activity after incubation in 5 M NaCl for 10 h at 4 °C. When acting on crystalline α-chitin, the main products of the three chitinases are all (GlcNAc)_2_, which suggests that chitinases Chia4287, Chib0431, and Chib0434 likely have potential in direct conversion of crystalline chitin into (GlcNAc)_2_.

## 1. Introduction

Chitin is a polymer of *N*-acetyl-d-glucosamine (GlcNAc) and is the second most abundant polysaccharide after cellulose in nature. Chitin is mainly present in arthropod exoskeletons, fungal cell walls, and insect cuticles in a crystalline form, which is intractable, highly hydrophobic, and insoluble in water [1]. Chitin has three polymorphic isomers, including α-chitin, β-chitin, and γ-chitin. Among them, α-chitin is the most common form found in fungi, insect exoskeletons, and shells of crustaceans. α-chitin is harder to degrade than β-chitin and γ-chitin as it has a higher degree of recalcitrance, which decreases the accessibility of the individual polymer chains [2]. Colloidal chitin is normally prepared by treating natural chitin with strong acids to break the crystal structure and increase the accessibility of the substrate to enzymes. Therefore, colloidal chitin is usually used as the substrate for chitinase characterization.

The annual production of chitin in the ocean exceeds billions of tons [3,4], which is a good source for the production of chitooligosaccharides (COSs) and GlcNAc. Due to their various bioactive activities, COSs and GlcNAc have been widely applied in agriculture, medicine, cosmetics, and foods. For example, COSs have protective effects against infections and enhanced antitumor properties [5,6]. GlcNAc and (GlcNAc)_2_ (*N*,*N*′-diacetylchitobiose) can serve as cosmetic ingredients, dietary supplements, and osteoarthritis therapeutics [7,8,9].

The chitinolytic enzymes contain chitinases (EC 3.2.1.14), mainly from the GH18 and GH19 families, and β-*N*-acetylglucosaminidases (EC 3.2.1.52), mainly from the GH20 and GH3 families. While several β-*N*-acetylglucosaminidases have been reported to be active on chitin [10,11,12], the hydrolysis of chitin into COSs and/or GlcNAc is predominantly catalyzed by chitinases [13]. Chitinases include endochitinases and exochitinases, which are widely produced by bacteria [14], fungi [15], and plants [16], playing key roles in natural chitin degradation and recycling. Many bacteria-derived chitinases have been characterized, predominantly with colloidal chitin or chitooligosaccharides (or synthetic chitooligosaccharide analogs) as the substrate. Reported bacterial chitinases are mostly mesophilic enzymes with optimal temperatures at 40–60 °C [17,18,19,20,21,22,23,24,25]; only a few have been found to be cold-active enzymes, such as CHI II of *Glaciozyma antarctica* PI12 (15 °C) [26] and ChiA of *Pseudoalteromonas* sp. DL-6 (20 °C) [27]. The pH optima of bacterial chitinases are over a wide range. For example, chitinases from *Streptomyces chilikensis* RC1830 [24], *Pseudoalteromonas tunicata* CCUG 44952^T^ [25], and *Bacillus* sp. R2 [21] showed their highest activity at neutral pHs (7.0–7.5), those from *Micrococcus* sp. AG84 [22], *Pseudoalteromonas* sp. DC14 [23], and *Citrobacter freundii* haritD11 [28] at basic pHs (8.0–9.0), and those from *Moritella marina* ATCC 15381 [29] and *Paenicibacillus barengoltzii* CAU904 [17] at acidic pHs (3.5 and 5.0, respectively). Chitinases are good tools to prepare COSs and GlcNAc from chitin. Because the natural source of chitin is crystalline chitin, chitinases that can efficiently hydrolyze crystalline chitin have better application potential in preparing COSs and GlcNAc from natural chitin sources than those only active on colloidal chitin. However, so far, only a few crude enzymes produced by wild strains and recombinant chitinases have been reported to be used in preparing COSs and GlcNAc from crystalline chitin [27,30,31,32,33]. Thus, it is necessary to identify and characterize more chitinases that can efficiently hydrolyze crystalline chitin for preparing COSs and GlcNAc from natural chitin sources.

Bacteria of the genus *Pseudoalteromonas* are widely distributed in the ocean, accounting for 2–3% of total bacterial abundance in upper ocean waters [34,35]. Many strains in this genus contain multiple chitinase-encoding genes [36], and some have been reported to secrete chitinases [18,23,25,27,37,38]. Furthermore, some chitinases from *Pseudoalteromonas* have been characterized. The GH18 chitinase Chi23, from *Pseudoalteromonas aurantia* DSM6057, is a thermostable enzyme with activity towards crystalline chitin in acidic conditions (pH 3.0–6.0) [18]. The GH18 chitinases ChiA and ChiC from *Pseudoalteromonas* sp. DL-6 [27,37] and ChiB from *Pseudoalteromonas* sp. O-7 [38] are cold-active enzymes with temperature optima at 20–30 °C. The GH19 chitinase Ptchi19 from *Pseudoalteromonas tunicata* CCUG 44952^T^ was active at 20–50 °C and pH 6.0–9.5 [25]. The chitinase purified from the fermentation broth of *Pseudoalteromonas* sp. DC14 exhibited halo-alkali and thermo-stable properties [23]. Despite these studies, *Pseudoalteromonas* chitinases with potential in preparing COSs/GlcNAc from natural crystalline chitin have rarely been reported. The aim of this study is to identify and characterize chitinases with activity on crystalline chitin from marine *Pseudoalteromonas* bacteria and to evaluate their potential in preparing COSs/GlcNAc from natural crystalline chitin. In this study, the ability of 26 *Pseudoalteromonas* type strains to use crystalline chitin as a carbon source for growth was investigated, and *Pseudoalteromonas flavipulchra* DSM 14401^T^ (hereafter strain DSM 14401), which was isolated from surface seawater [39], was found to have the highest degradation rate on crystalline α-chitin. The extracellular chitinases secreted by strain DSM 14401 were further identified by genome and secretome analyses. Three chitinases with high abundance in the secretome were heterologously expressed in *Escherichia coli* BL21 (DE3) and biochemically characterized. The hydrolytic products released from crystalline chitin by these chitinases were further investigated. The results suggest that these chitinases likely have potential in the preparation of (GlcNAc)_2_ from natural crystalline chitin.

## 2. Results and Discussion

### 2.1. The Ability of Strain DSM 14401 to Utilize Crystalline Chitin

**Figure 1:**
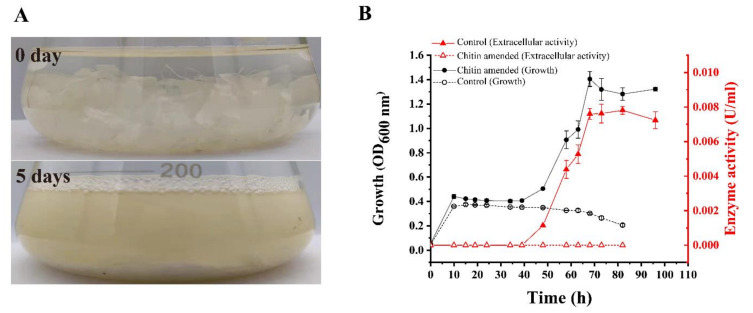
Growth and extracellular chitinase activity of *P. flavipulchra* DSM 14401^T^ cultured on crystalline chitin. (**A**) Cultures of strain DSM 14401 at 25 °C for 0 and 5 days. (**B**) The growth curve (black line) and the extracellular chitinase activity (red line) of strain DSM 14401. Strain DSM 14401 was cultured in a minimal medium containing 0.05% peptone, 0.01% yeast powder, and 3% (*w*/*v*) chitin flakes at 25 °C and 180 rpm. The extracellular chitinase activity was measured with chitin powder as the substrate at 50 °C. Strain DSM 14401 cultured in the same medium without chitin flakes and under the same conditions was used as the control.

To obtain *Pseudoalteromonas* strains that can secrete chitinases to efficiently degrade crystalline chitin, 26 type *Pseudoalteromonas* strains (Table S1) were cultured in a liquid medium containing chitin flakes (crystalline α-chitin) as carbon source, and their growth and the degree of degradation of chitin flakes were observed. Strain DSM 14401 showed the greatest degradation rate of chitin flakes. This strain was able to degrade most of the chitin flakes in the medium in 5 days (Figure 1A). The growth curve and the extracellular chitinase activity of strain DSM 14401 during cultivation were also investigated (Figure 1B). The strain was cultured in a medium containing 0.05% peptone, 0.01% yeast powder, and 3% chitin flakes; the same medium without chitin flakes was used as a control. Strain DSM 14401 grew rapidly in the first 10 h in both media, with or without chitin flakes. After 10 h, the growth stagnated in both media, likely due to the depletion of the absorbable nutrients, such as peptone and yeast powder. After 40 h, while the cell number in the control medium began to decrease slowly and no extracellular chitinase activity was detected during the cultivation, both the cell number and the extracellular chitinase activity in the medium containing chitin flakes began to continuously increase until 68 h (Figure 1B). Based on this result, it can be speculated that, after absorbable nutrients were depleted, strain DSM 14401 began to secrete chitinases to degrade the chitin flakes in the medium into COSs/GlcNAc, which were absorbed by the strain to support its growth.

Paulsen et al. reported that 27 *Pseudoalteromonas* strains have the ability to degrade crystalline chitin [36]. Strain *Pseudoalteromonas* sp. DC14 was also reported to be able to degrade crystalline chitin [23]. In addition, 5 chitinases from *Pseudoalteromonas* strains have been expressed and characterized, including ChiA and ChiC from *Pseudoalteromonas* sp. DL-6 [27,37], ChiB from *Pseudoalteromonas* sp. O-7 [38], PtChi19p from *P. tunicata* CCUG 44952^T^ [25] and Chi23 from *P. aurantia* DSM6057 [18]. Among them, chitinases ChiA, PtChi19p, and Chi23 have activity on crystalline chitin based on substrate specificity analysis [18,25,27]. These reports indicate that many *Pseudoalteromonas* strains can produce chitinases with activity on crystalline chitin. Consistently, strain DSM 14401 was most likely to secrete chitinases with activity on crystalline chitin due to its high degradation rate on crystalline α-chitin.

### 2.2. Identification of the Chitinases Secreted by Strain DSM 14401

**Figure 2:**
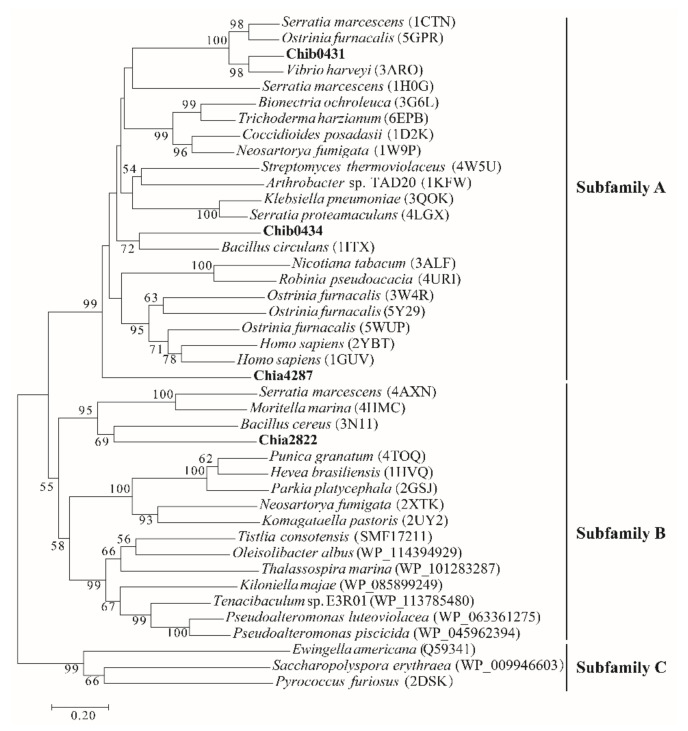
Phylogenetic analysis of chitinases Chib0431, Chib0434, Chia4287, and Chia2822 with other GH18 chitinases. The phylogenetic tree was constructed by the Neighbor-Joining method. Bootstrap analysis of 1000 replicates was conducted.

**Figure 3:**
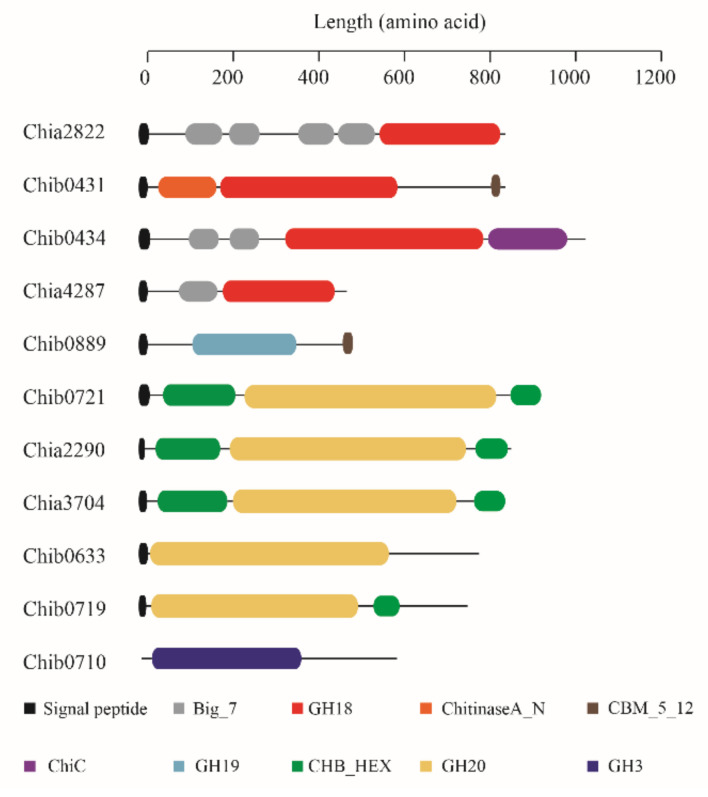
Domain architecture of the 11 chitinolytic enzymes of *P. flavipulchra* DSM 14401^T^. Protein sequences were analyzed on the HMMER website, and domains were illustrated by different colors based on their functional annotations. The Pfam IDs corresponding to the function annotations are as follows: Big_7, bacterial Ig domain (PF17957); GH18, glycosyl hydrolases family 18 (PF00704); ChitinaseA_N, ChitinaseA_N-terminal domain (PF08329); CBM_5_12, carbohydrate-binding module (PF02839), ChiC, Chitinase C (PF06483); GH19, glycoside hydrolase family 19 (PF00182), CHB_HEX, putative carbohydrate-binding domain (PF03173); GH20, glycosyl hydrolase family 20 (PF00728); GH3, glycosyl hydrolase family 3 (PF00933).

To ascertain the chitinolytic enzymes secreted by strain DSM 14401, genomic analysis was carried out to find putative chitinolytic enzyme-encoding genes in strain DSM 14401. There are 11 genes encoding putative chitinolytic enzymes in strain DSM 14401, which were named Chia2822, Chib0431, Chib0434, Chia4287, Chib0889, Chib0721, Chia2290, Chia3704, Chib0633, Chib0719 and Chib0710. Chia2822, Chib0431, Chib0434, and Chia4287 are potential chitinases belonging to the GH18 family (Figure 2). Of these, Chib0431, Chib0434, and Chia4287 belong to the GH18A subfamily that mainly contains processive exochitinases [40,41,42], and Chia2822 belongs to the GH18B subfamily that mainly contains non-processive endochitinases [43,44]. Multiple sequence alignments suggest that all these GH18 chitinases of strain DSM 14401 contain a DxDxE catalytic motif (Figure S1), which is conserved in the GH18 chitinases [45]. Chitinase Chib0889 belongs to the GH19 family that mainly contains chitinases found in plants [46]. Two GH19 chitinases, LYS177 and LYS188, from *Pseudomonas* Ef1 have been reported to have lysozyme activity and they are clustered with phage/prophage endolysins based on the phylogenetic analysis [47]. However, the GH19 chitinase, Chib0889, of strain DSM 14401 was nested in the cluster of chitinases from Proteobacteria (Figure S2), implying that Chib0889 may function as a chitinase rather than a lysozyme. Chib0721, Chia2290, Chia3704, Chib0633, and Chib0719 from the GH20 family, and Chib0710 from the GH3 family are potential β-*N*-acetylglucosaminidases. The predicted domain architectures of these chitinolytic enzymes are shown in Figure 3. Except for Chib0710, the other chitinolytic enzymes all have a signal peptide predicted by SignalP 5.0, implying that they are likely secreted enzymes. Among these enzymes, Chib0633 and Chib0710 are single-domain enzymes, while the others are all multi-domain enzymes containing one or more carbohydrate-binding domains (Big_7, CBM_5_12, and CHB_HEX) in addition to their catalytic domains. The CBMs (carbohydrate-binding modules) in chitinases were reported to facilitate enzyme movement along a chitin chain during processive action and to stimulate the substrate to decrystallize [48,49,50,51].

Secretome analysis was further performed to identify the chitinolytic enzymes secreted by strain DSM 14401 cultured in the medium containing 3% chitin flakes as the sole carbon source. The extracellular proteins tightly absorbed on the chitin flakes were collected for secretome analysis when approximately half of the chitin flakes in the medium were degraded after 85 h. Finally, 6 of the putative chitinolytic enzymes were detected in the secretome. Of these, the 4 GH18 chitinases accounted for 97.50% of the abundance, and the GH19 and GH20 chitinolytic enzymes each accounted for 1.25% (Table 1), which suggests the importance of the GH18 chitinases in the degradation of crystalline chitin. Of the GH18 chitinases, Chia4287 was the most abundant (48.75%), followed by Chib0431 (25.00%), Chib0434 (15.00%) and Chia2822 (8.75%). The five putative β-*N*-acetylglucosaminidases with a predicted signal peptide were not found in the secretome, which may be secreted to the periplasm.

It has been reported that chitinolytic strains belonging to the genus *Pseudoalteromonas* usually have two GH18 chitinase genes in their chitin degradation clusters [36]. In addition, many *Pseudoalteromonas* species also contain one or more GH19 chitinase genes [36]. However, the removal of the GH19 chitinase gene from strain *Pseudoalteromonas* *rubra* S4059 had no significant influence on the growth of the strain on crystalline α-chitin [52], suggesting that the GH19 chitinase is likely unimportant in the utilization of crystalline chitin. In contrast, the removal of the GH18 chitinase gene *chiD* from strain *Cellvibrio japonicus* Ueda107 made it unable to grow on crystalline α-chitin [53], indicating that the GH18 chitinase plays an important role in the crystalline chitin degradation of this strain. Moreover, it has been reported that (GlcNAc)_2_ and larger chitooligosaccharides can induce the expression of chitinases in *Vibrio furnissii* 7225 and *Vibrio cholerae* O1 [54]. For strain DSM 14401, although its genome contains a GH19 chitinase gene, a GH3 β-*N*-acetylglucosaminidase gene, and 5 GH20 β-*N*-acetylglucosaminidase genes in addition to 4 GH18 chitinase genes, secretome analysis showed that it mainly secreted the GH18 chitinases when crystalline α-chitin was present, which suggests that the GH18 chitinases likely play a main role in the degradation of crystalline α-chitin in this strain.

### 2.3. Characterization of the GH18 Chitinases with Activity on Crystalline Chitin

**Figure 4:**
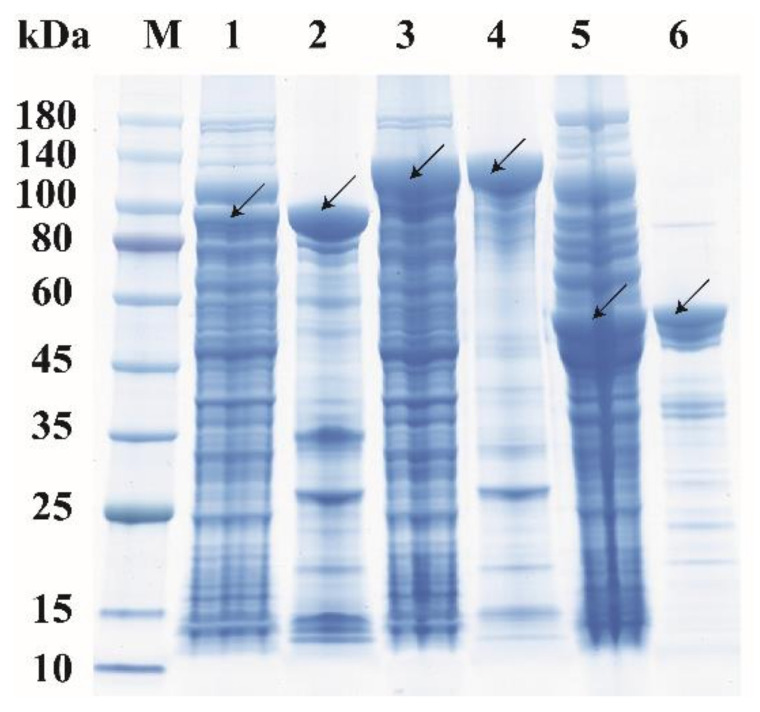
The SDS-PAGE analysis of recombinant proteins Chib0431, Chib0434, and Chia4287. Lane M, protein molecular mass marker; Lane 1, the cell lysate of *E. coli* containing recombinant protein Chib0431; Lane 2, the purified recombinant protein Chib0431; Lane 3, the cell lysate of *E. coli* containing recombinant protein Chib0434; Lane 4, the purified recombinant protein Chib0434; Lane 5, the cell lysate of *E. coli* containing recombinant protein Chia4287; Lane 6, the purified recombinant protein Chia4287. The enzyme bands are indicated by arrows.

The high abundance of the GH18 chitinases in the secretome of strain DSM 14401 implies that they are likely to be the chitinases with activity on crystalline chitin. Thus, 3 GH18 chitinases, Chia4287, Chib0431, and Chib0434, with high abundance in the secretome, were selected to be expressed and characterized. Genes encoding Chia4287, Chib0431, and Chib0434 were heterologously expressed in *E. coli* BL21 (DE3), and the recombinant proteins were purified by NTA-Ni Sepharose affinity chromatography (Figure 4). The purification folds for Chib0431, Chib0434 and Chia4287 were 6.75, 5.33, and 7.30, respectively (Table S2). As shown in Figure 4, the 3 purified recombinant proteins have apparent molecular weights of approximately 88 kDa (Chib0431), 112 kDa (Chib0434), and 51 kDa (Chia4287), consistent with their theoretical molecular weights (Table 1).

To investigate the substrate specificity of these 3 chitinases, the enzyme activities of Chib0431, Chib0434, and Chia4287 toward colloidal chitin, chitin powder, chitosan, microcrystalline cellulose, 4-Methylumbelliferyl *N*-acetyl-β-D-glucosaminide (MUF-GlcNAc) [55], 4-Methylumbelliferyl-β-D-*N*,*N*′-diacetylchitobioside hydrate (MUF-(GlcNAc)_2_) [56], and 4-Methylumbelliferyl-β-D-*N*,*N*′,*N*″-triacetylchitotrioside (MUF-(GlcNAc)_3_) [57] were determined. As shown in Table 2, all the three chitinases had activity toward colloidal chitin, crystalline chitin, MUF-(GlcNAc)_2,_ and MUF-(GlcNAc)_3_, but neither had activity toward chitosan, microcrystalline cellulose, or MUF-GlcNAc. Among them, Chia4287 had the highest activity towards chitin powder, followed by Chib0431 and Chib0434, which is consistent with their amount in the secretome. Chitinases Chia4287 and Chib0431 exhibited higher activities toward MUF-(GlcNAc)_3_ than MUF-(GlcNAc)_2_, suggesting that both enzymes likely function as endochitinases. In contrast, Chib0434 showed approximately 10-fold higher activity toward MUF-(GlcNAc)_2_ than MUF-(GlcNAc)_3_, suggesting that Chib0434 tends to act as an exochitinase.

**Figure 5:**
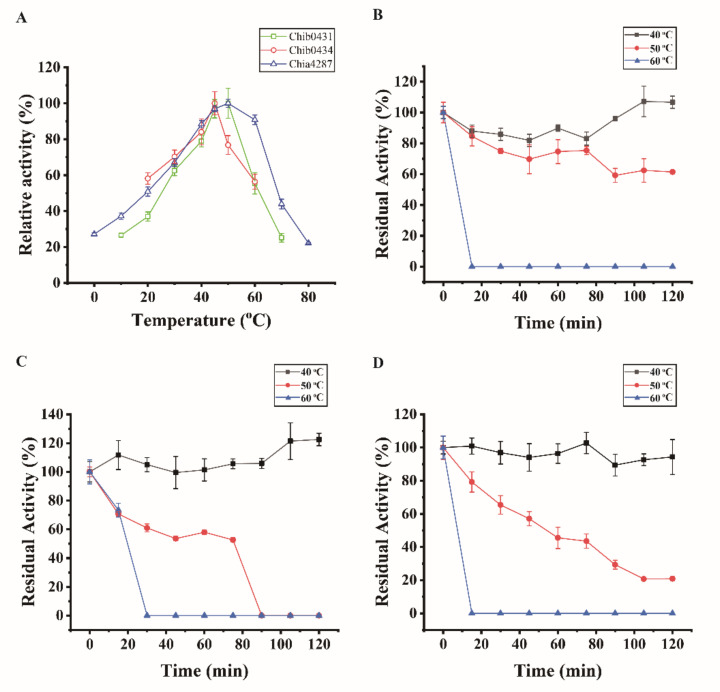
Effect of temperature on the activities and stabilities of chitinases Chib0431, Chib0434, and Chia4287. (**A**) Effect of temperature on the activities of Chib0431, Chib0434, and Chia4287. The activities of each enzyme were measured at its optimal pH with chitin powder as the substrate. The highest activity of each enzyme was defined as 100%. (**B**) Effect of temperature on the stability of Chib0431. (**C**) Effect of temperature on the stability of Chib0434. (**D**) Effect of temperature on the stability of Chia4287. In B, C, and D, the residual activities of each enzyme were measured at its optimal temperature and pH with chitin powder as the substrate, and the activity of each enzyme without incubation was defined as 100%. The graphs show data from triplicate experiments (mean ± SD).

**Figure 6:**
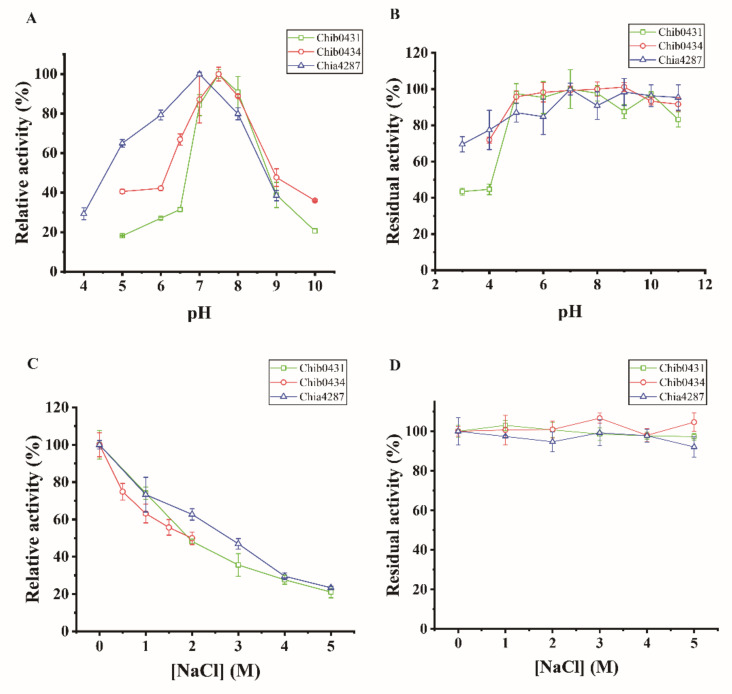
Effects of pH and NaCl on the activities and stabilities of chitinases Chib0431, Chib0434, and Chia4287. (**A**) Effect of pH on the activities of Chib0431, Chib0434, and Chia4287. The activities of each enzyme were measured at its optimal temperature with chitin powder as the substrate. The highest activity of each enzyme was defined as 100%. (**B**) Effect of pH on the stabilities of Chib0431, Chib0434, and Chia4287. The residual activities of each enzyme were measured at its optimal temperature and pH with chitin powder as the substrate. (**C**) Effect of NaCl concentration on the activities of Chib0431, Chib0434, and Chia4287. The activities of each enzyme were measured at its optimal temperature and pH with chitin powder as the substrate. The activity of each enzyme in 0 M NaCl was defined as 100%. (**D**) Effect of NaCl concentration on the stabilities of Chib0431, Chib0434, and Chia4287. The residual activities of each enzyme were measured at its optimal temperature and pH with chitin powder as the substrate. The highest activity of each enzyme was defined as 100%. The graphs show data from triplicate experiments (mean ± SD).

With chitin powder as the substrate, the three chitinases were biochemically characterized. Both chitinases Chib0431 and Chia4287 showed optimum temperatures at 50 °C, and Chib0434 at 45 °C (Figure 5A). For their thermal stability, Chib0431 retained approximately 100% activity at 40 °C and more than 61% at 50 °C after 120 min incubation but lost all its activity at 60 °C in 15 min (Figure 5B). Chib0434 retained 100% activity at 40 °C after 120 min incubation but lost all its activity at 50 °C in 90 min and at 60 °C in 30 min (Figure 5C). Chitinase Chia4287 retained high activity (≥89%) when incubated at 40 °C for 120 min (Figure 5D). Chitinases Chib0434 and Chia4287 both showed highest activity at pH 7.5 and Chib0431 at pH 7.0 (Figure 6A). For their pH stability, the 3 chitinases all exhibited high stability (retaining ≥80% activity) from pH 5.0 to 11.0 in the Britton–Robinson buffer after 10 h incubation at 4 °C (Figure 6B). They all showed highest activity at 0 M NaCl (Figure 6C) but maintained high activity (≥92%) in 1–5 M NaCl after 10 h incubation at 4 °C (Figure 6D). Therefore, the 3 chitinases have temperature optima of 45–50 °C and pH optima of 7.0–7.5, indicating that they are all neutral and mesophilic enzymes. They are all stable at 40 °C and in the pH range of 5.0–11.0, and all have excellent salt tolerance.

Many chitinases have been heterologously expressed and characterized with colloidal chitin or synthetic chitooligosaccharide analogs. As shown in Table 3, the temperature and pH optima of the reported chitinases and their thermostability are quite diverse. So far, several *Pseudoalteromonas* GH18 chitinases have been characterized (Table 3). The chitinase Chi23 from *P. aurantia* DSM6057 was reported to be thermostable but active toward crystalline chitin only in acidic conditions (pH of 3.0–6.0) [18]. Chitinases ChiA and ChiC from *Pseudoalteromonas* sp. DL-6 [27,37] and ChiB from *Pseudoalteromonas* sp. O-7 [38] are all cold-active enzymes with optimal activities at 20–30 °C and low thermostability. The 3 mesophilic chitinases, Chib0431, Chib0434, and Chia4287, characterized in this study are active toward crystalline chitin at neutral pH conditions (pH 7.0–7.5) and have good thermostability and pH- and salt-tolerance, which, therefore, may be good candidates for industrial application.

### 2.4. Analysis of the Products of the Chitinases on Crystalline Chitin

**Figure 7:**
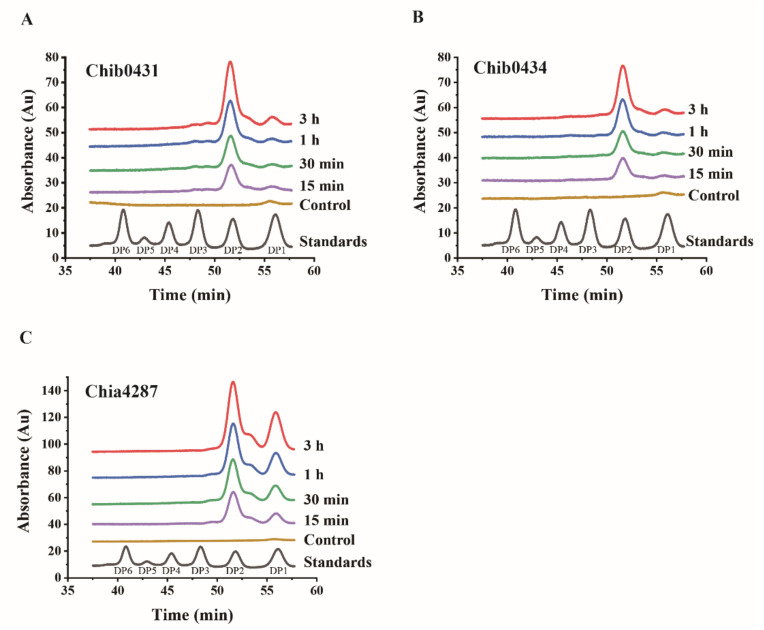
Analysis of the degradation products of the three chitinases on crystalline chitin. (**A**) The degradation product of Chib0431. (**B**) The degradation product of Chib0434. (**C**) The degradation product of Chia4287. Chitin powder was degraded by the chitinases at their respective optimal temperatures for different times (15 min, 30 min, 1 h, and 3 h). The reaction system with enzyme inactivated at 100 °C for 10 min was used as the control. The reaction was terminated by boiling at 100 °C for 10 min, and then the reaction mixtures were centrifuged at 17,949× *g* for 10 min. The products in the supernatants were analyzed by gel filtration chromatography on a Superdex Peptide10/300 GL column (GE Healthcare, Sweden), which were monitored at a wavelength of 210 nm. The injected volume was 10 μL. DP1-DP6 are chitooligosaccharide markers. DP1, GlcNAc; DP2, (GlcNAc)_2_; DP3, (GlcNAc)_3_; DP4, (GlcNAc)_4_; DP5, (GlcNAc)_5_; DP6, (GlcNAc)_6_.

In order to investigate the application potential of the three chitinases in preparing COSs/GlcNAc from natural chitin, we analyzed the degradation products of Chia4287, Chib0431, and Chib0434 towards crystalline chitin. The reaction mixtures, containing chitin powder and chitinases, were incubated at their respective optimal temperatures for different time periods (15 min, 30 min, 1 h, and 3 h). The COSs/GlcNAc released from chitin in the supernatants of the mixtures were analyzed by gel filtration chromatography on a Superdex Peptide 10/300 GL column. For Chib0431 and Chib0434, during the 3 h degradation of crystalline chitin, (GlcNAc)_2_ was always the predominant product, with only a slight amount of GlcNAc (Figure 7A,B). However, in the hydrolytic products of Chia4287 on crystalline chitin, although (GlcNAc)_2_ was also the main product, the proportion of GlcNAc was much higher compared to that in the hydrolytic products of Chib0431 and Chib0434 (Figure 7C). Together, these results indicate that Chia4287, Chib0431, and Chib0434 can degrade crystalline chitin into (GlcNAc)_2_ and GlcNAc, with (GlcNAc)_2_ as the main product. These results imply that they may have potential in the preparation of (GlcNAc)_2_ from natural crystalline chitin.

COSs/GlcNAc have been widely prepared with a variety of crude enzymes from wild strains and purified recombinant chitinases, most of which were prepared with colloidal chitin [17,58,59,60,61]. So far, however, there have been only a few chitinases used to prepare COSs/GlcNAc from natural crystalline chitin. The enzyme cocktail of strain *Paenibacillus* sp. LS1 can produce GlcNAc and (GlcNAc)_2_ with minor (GlcNAc)_3_ from crystalline α-chitin [30]. The crude enzyme of *Aeromonas hydrophila* H-2330 mainly produces GlcNAc from crystalline α-chitin [31]. The chitinase ChiA of strain *Pseudoalteromonas* sp. DL-6 is an endochitinase, and its products on crystalline α-chitin are a mixture of chitin COSs (DP 2–6), with (GlcNAc)_2_ as the major product [27]. The mixture of purified chitinases SaChiB and SaHEX of strain *Streptomyces alfalfa* ACCC40021 can enhance the conversion of crystalline α-chitin to GlcNAc [62]. The chitinase of strain *Chitinibacter* sp. GC72 can degrade practical-grade chitin into GlcNAc [33]. The three chitinases characterized in this study can degrade crystalline α-chitin into (GlcNAc)_2_, suggesting their potential in direct conversion of natural crystalline chitin into (GlcNAc)_2_.

## 3. Materials and Methods

### 3.1. Bacterial Strains and Experimental Materials

The 26 type strains of genus *Pseudoalteromonas* were purchased from Deutsche Sammlung von Mikroorganismen and Zelkulturen (DSMZ) or Japan Collection of Microorganisms (JCM). Chitin powder (crystalline α-chitin), MUF-GlcNAc, MUF-(GlcNAc)_2,_ and MUF-(GlcNAc)_3_ were purchased from Sigma-Aldrich (St. Louis, MO, USA). Chitin flakes, purchased from Yuan Cheng Group (Wuhan, China), are crystalline α-chitin. Colloidal chitin was prepared as previously described [18]. GlcNAc, (GlcNAc)_2_, (GlcNAc)_3_, (GlcNAc)_4_, (GlcNAc)_5,_ and (GlcNAc)_6_ were purchased from BZ Oligo Biotech Co., LTD (Qingdao, China). Chitosan was purchased from Sangon Biotech (Shanghai, China). BCA protein assay kit was purchased from Thermo Scientific (Boston, MA, USA). Other chemicals were of analytical grade and commercially available.

### 3.2. Screening of Strain DSM 14401

The 26 type strains of genus *Pseudoalteromonas* (Table S1) were cultivated at 25 °C and 180 rpm in the TYS medium composed of 0.5% (*w*/*v*) peptone, 0.1% (*w*/*v*) yeast powder, and artificial seawater (pH 7.8). When the OD_600_ of the culture was approximately 1.0, 2 mL cell suspension was collected and the cells were washed with the minimal medium (30 g/L NaCl, 0.5 g/L NH_4_Cl, 3 g/L MgCl_2_·6H_2_O, 2 g/L K_2_SO_4_, 0.2 g/L K_2_HPO_4_, 0.01 g/L CaCl_2_, 0.006 g/L FeCl_3_·6H_2_O, 0.005 g/L Na_2_MoO_4_·7H_2_O, 0.004 g/L CuCl_2_·2H_2_O, 6 g/L Tris, pH 7.6) three times. Then, the washed cells were inoculated into the minimal medium supplemented with 0.05% (*w*/*v*) peptone, 0.01% (*w*/*v*) yeast powder, and 3% (*w*/*v*) chitin flakes and cultivated at 25 °C and 180 rpm for 5 days. Their growth and the degree of degradation of the chitin flakes were observed every day. Among them, strain DSM 14401 showed the highest degradation rate on crystalline α-chitin, which was then chosen for further study. The OD_600_ of the culture of this strain in the medium was measured at different time intervals, as indicated in Figure 1, to produce its growth curve. The washed cells were cultured in the same medium without chitin flakes and in the same conditions as the control.

### 3.3. Extracellular Chitinase Activity Assay of Strain DSM 14401

During the cultivation of strain DSM 14401 in the above liquid medium with or without chitin flakes, 1 mL of culture was taken out at different intervals, as indicated in Figure 1. The cultures were filtered with a 0.22 μm filter to remove the bacterial cells, and the filtrate was used for the extracellular chitinase activity assay. A 200 μL mixture consisting of 50 mM Tris-HCl (pH 7.0), 3% chitin powder, and 50 μL of filtrate was incubated at 50 °C for 2 h. The mixture was then centrifuged at 17,949× *g* for 2 min at 4 °C and the supernatant obtained was used for the reducing-sugar assay by the DNS method [63]. The control mixture contained a pre-boiled filtrate instead of the filtrate. Subsequently, the optical density at 550 nm was measured to quantify the released reducing sugar. The amount of reducing sugar generated was calculated using GlcNAc as a standard. One unit of enzyme activity was defined as the amount of enzyme that liberated 1 μmol of reducing sugar per minute.

### 3.4. Bioinformatics Analysis

The genome DNA of strain DSM 14401 was sequenced by our lab [64]. The putative chitinases of this strain were determined according to dbCAN [65] analyses. Signal peptides of the chitinases were predicted by SignalP 5.0 (http://www.cbs.dtu.dk/services/SignalP/ (accessed on 12 January 2022)) [66]. The domain architectures of the chitinases were predicted on the HMMER website (https://www.ebi.ac.uk/Tools/hmmer/search/hmmscan (accessed on 12 January 2022)) [67]. The phylogenetic tree was constructed based on the Neighbor-Joining method and using the Poisson model with MEGA X after multiple alignments of the sequences by MUCLE [68]. Sequences alignment results were visualized using the ESPript 3.0 server [69]. The molecular weights of the chitinases were predicted by the ExPASy Server (https://web.expasy.org/compute_pi/ (accessed on 12 January 2022)) [70].

### 3.5. Secretome Analysis

Strain DSM 14401 was cultured at 25 °C and 180 rpm in a medium containing the minimal medium and 3% chitin flakes. When approximately half of the chitin flakes were degraded, the culture was centrifuged at 8228× *g* at 4 °C for 6 min. The precipitates were resuspended using 20 mM Tris-HCl (pH 8.0) containing 1 M NaCl, and then centrifuged at 1157× *g* at 4 °C for 3 min. This step was repeated three times. The resultant precipitates were resuspended using 50 mM Tris-HCl (pH 8.0) containing 6 M Guanadine-HCl, and then centrifuged at 15,557× *g* at 4 °C for 10 min. The supernatant was moved into an ultrafiltration tube (15 mL, 3 kDa). The Guanadine-HCl in the supernatant was removed by adding 50 mM Tris-HCl to the ultrafiltration tube (molecular weight cut-off, 3 kDa) and centrifugation (4629× *g* for 10 min at 4 °C) for three times. Then, the proteins in the supernatant were precipitated by 50 mL acetone containing 10% trichloroacetic acid and 0.1% dithiothreitol overnight at −20 °C. The precipitates were harvested and washed by 80% acetone and 100% acetone successively, and then lyophilized. The lyophilized sample was successively denatured, reduced, and alkylated by denaturation buffer (0.5 M Tris-HCl, 2.75 mM EDTA, 6 M Guanadine-HCl), dithiothreitol (1 M), and iodoacetamide (1 M), respectively. The sample solution was further replaced with 25 mM NH_4_HCO_3_ solution by centrifugation ultrafiltration (15,294× *g* for 15 min at 4 °C) in an ultrafiltration tube (1 mL, 3 kDa). The sample was digested using trypsin at 37 °C for 12 h, and the resultant peptides were desalted on a C_18_ column (ZipTip C18, Millipore, Billerica, MA, USA). The desalted peptides were analyzed using the mass spectrometer Orbitrap Elite (Thermo Fisher Scientific, Bremen, Germany) coupled with Easy-nLC 1000 (Thermo Fisher Scientific, Bremen, Germany). Finally, the raw data was analyzed against the genome of strain DSM 14401 using Thermo Scientific Proteome Discoverer^TM^ 1.4. The mass spectrometry proteomics data have been deposited to the ProteomeXchange [71] Consortium via the PRIDE [72] partner repository with the dataset identifier PXD030600. The reviewer account details: Username: reviewer_pxd030600@ebi.ac.uk; Password: 1QCP2jqI.

### 3.6. Expression and Purification of Chitinases Chib0431, Chib0434, Chia4287

The gene sequences of Chib0431, Chib0434, and Chia4287 without the signal peptide were cloned from the genomic DNA of strain DSM 14401 and inserted into the NdeI and XhoI sites of the expression vector pET-22b(+). The constructed recombinant plasmids were then transformed into *E. coli* BL21(DE3) for protein expression. The constructed recombinant *E. coli* BL21(DE3) strains were cultured at 37 °C in liquid LB medium containing 100 μg/mL ampicillin. When the OD_600_ of the cultures reached 0.6–1.0, 0.45 mM isopropyl thio-β-D-galactoside (IPTG), used as an inducer, was added into the cultures, and the cultures were incubated at 18 °C for 16 h. Then, the recombinant *E. coli* cells in the cultures were collected via centrifugation and crushed by sonication in the lysis buffer (100 mM NaCl, 5 mM imidazole, 50 mM Tris-HCl pH 8.0). The recombinant proteins of Chib0431, Chib0434, and Chia4287 in the cell extracts were further purified by affinity chromatography with Ni-NTA agarose resins (Qiagen, Santa Clarita, CA, USA), followed by desalination on PD-10 Desalting Columns (GE Healthcare, Piscataway, NJ, USA), using 10 mM Tris-HCl containing 100 mM NaCl (pH 8.0) as the running buffer. The purified proteins were analyzed by sodium dodecyl sulfate polyacrylamide gel electrophoresis (SDS-PAGE) [73]. The protein concentrations were determined using a BCA protein assay kit with bovine serum albumin (BSA) as the standard.

### 3.7. Enzyme Assays

The activities of the three purified chitinases towards chitin powder, colloidal chitin, chitosan, microcrystalline cellulose, MUF-GlcNAc, MUF-(GlcNAc)_2_ and MUF-(GlcNAc)_3_ were assayed in 50 mM Tris-HCl at their respective optimal temperatures and pHs (50 °C and pH 7.0 for Chia4287, 50 °C and pH 7.5 for Chib0431, 45 °C and pH 7.5 for Chib0434). When the insoluble chitin powder, chitosan, or microcrystalline cellulose was used as the substrate, the reaction mixture contained 190 µL 50 mM Tris-HCl, 3% (*w*/*v*) substrate and 10 µL enzyme, which was incubated for 1 h for Chia4287 or 2 h for Chib0431 and Chib0434. When colloidal chitin was used as the substrate, the reaction mixture contained 190 µL 0.75% (*w*/*v*) colloidal chitin in 50 mM Tris-HCl and 10 µL enzyme, which was incubated for 40 min. After incubation, the activities of the chitinases towards these substrates were determined using the DNS method [63]. The enzyme activity (U) was defined as the amount of enzyme that required to release 1 μmol GlcNAc equivalent reducing sugar from the substrate per minute. When MUF- GlcNAc, MUF-(GlcNAc)_2_, or MUF-(GlcNAc)_3_ was used as the substrate, the enzyme activity was assayed for 15 min with the reaction mixture contained 790 µL 1 mM substrate in 50 mM Tris-HCl and 10 µL enzyme, which was incubated for 15 min and then terminated by an addition of 0.4 M NaCO_3_. The enzyme activity (U) was defined as the amount of enzyme that required to release 1 μmol MUF from the substrate per minute.

### 3.8. Characterization of the Chitinases

The purified Chib0431, Chib0434, and Chia4287 were characterized with chitin powder as substrate. The effect of temperature on the enzyme activity was measured by assaying the enzyme activity at different temperatures (0–80 °C for Chia4287; 10–70 °C for Chib0431 and 20–60 °C for Chib0434) and their respective optimal pHs. The effect of pH on the enzyme activity was measured by assaying the enzyme activity in the Britton-Robinson buffer at different pHs (pH 4.0–9.0 for Chia4287; pH 5.0–10.0 for Chib0431 and Chib0434) and their respective optimal temperatures. Effect of salinity on the enzyme activity was assayed by assaying the enzyme activity in 50 mM Tris-HCl containing different concentrations of NaCl (0–5 M for Chib0431 and Chia4287; 0–2 M for Chib0434) at their respective optimal temperatures and pHs.

For the thermal stability assay, the purified chitinases were incubated at 40 °C, 50 °C, or 60 °C for 0–120 min, and the residual activities towards chitin powder were measured at an interval of 15 min under their respective optimal temperatures and pHs. For the pH stability assay, the purified chitinases were incubated in the Britton-Robinson buffers ranging from pH 3.0 to pH 11.0 at 4 °C for 10 h, and the residual activities towards chitin powder were measured at their respective optimal temperatures and pHs. For the halotolerance assay, the purified chitinases were incubated in 50 mM Tris-HCl containing different concentrations of NaCl (0–5 M) at 4 °C for 10 h, and the residual enzyme activities towards chitin powder were measured at their respective optimal temperatures and pHs.

### 3.9. Analysis of the Products Released from Crystalline Chitin by the Chitinases

The purified Chib0431, Chib0434, and Chia4287 (10 μL) were incubated with 3.0% chitin powder in 190 μL of 50 mM Tris-HCl (pH 7.0) for different times (15 min, 30 min, 1 h, and 3 h) at their respective optimal temperatures. The reaction was terminated by boiling at 100 °C for 10 min, and the reaction mixtures were centrifuged at 17,949× *g* for 10 min. Then, the products in the supernatants were analyzed by gel filtration chromatography on a Superdex Peptide 10/300 GL column (GE Healthcare, Uppsala, Sweden), which were monitored at 210 nm using a UV detector. The injected volume was 10 μL. The products were eluted with 0.2 M ammonium hydrogen carbonate for 90 min with a flow rate of 0.3 mL/min. The reaction system containing 10 μL enzyme pre-heated at 100 °C for 10 min was used as the control. A mixture of GlcNAc, (GlcNAc)_2_, (GlcNAc)_3_, (GlcNAc)_4_, (GlcNAc)_5_, and (GlcNAc)_6_ was used as the marker.

## 4. Conclusions

COSs have wide application in agriculture, medicine, cosmetics, and foods. While most COSs are now prepared with colloidal chitin, there are only a few reports of chitinases with potential in the preparation of COSs from natural crystalline chitin. In this study, three chitinases with activity on crystalline chitin were identified from a marine *Pseudoalteromonas* strain and characterized. These chitinases are all neutral mesophilic enzymes, which are most active at 45–50 °C and pH 7.0–7.5, and have high stability at 40 °C, pH 5.0–11.0, and in 5 M NaCl. The main products of the three chitinases on crystalline chitin are all (GlcNAc)_2_, suggesting that these chitinases have potential in preparing (GlcNAc)_2_ via direct degradation of natural crystalline chitin. Further studies such as improving the expression amount of these chitinases and their degradation efficiency on crystalline chitin are underway.

## Figures and Tables

**Table 1:** The extracellular chitinolytic enzymes secreted by strain DSM 14401 identified by secretome analysis.

Chitinolytic Enzyme	Accession Number	Family	Length (aa)	Molecular Weight (kDa)	PSMs ^a^	Abundance ^b^
Chia4287	WP_039494805	GH18	479	50.86	39	48.75%
Chib0431	WP_039495329	GH18	822	87.51	20	25.00%
Chib0434	WP_039495331	GH18	1037	112.17	12	15.00%
Chia2822	WP_039492151	GH18	850	90.42	7	8.75%
Chib0889	WP_084204324	GH19	470	53.05	1	1.25%
Chib0721	WP_039496328	GH20	915	101.45	1	1.25%

^a^ Peptide-Spectrum Matches. ^b^ Abundance was calculated based on the proportion of the PSMs of a chitinolytic enzyme in the sum of PSMs of all chitinolytic enzymes in the secretome.

**Table 2:** The substrate specificity of the three chitinases of strain DSM 14401 ^a^.

Substrate	Specific Activity (U/mg)		
	Chia4287	Chib0431	Chib0434
Colloidal chitin	0.53 ± 0.05	0.15 ± 0.04	0.09 ± 0.02
Chitin powder	0.17 ± 0.005	0.04 ± 0.002	0.01 ± 0.001
Chitosan	ND ^b^	ND	ND
Microcrystalline cellulose	ND	ND	ND
MUF-GlcNAc	ND	ND	ND
MUF-(GlcNAc)_2_	130.32 ± 3.29	19.69 ± 1.30	221.68 ± 12.15
MUF-(GlcNAc)_3_	139.33 ± 26.96	423.12 ± 9.82	23.47 ± 3.57

^a^ The data in the table are from three experiment repeats (mean ± SD). ^b^ ND means that the enzyme activity was not detectable.

**Table 3:** Characteristics of bacterial chitinases.

Enzyme	Family	Molecular Weight (kDa)	pH Optimum	Temperature Optimum (°C)	NaCl Optimum (M)	Thermostability (Half-Life)	Substrate (Specific Activity)	Hydrolytic Products (Substrate)	References
Chib0431 from *Pseudoalteromonas flavipulchra* DSM 14401^T^	GH18	87.51	7.5	50	0	>2 h at 50 °C	α-chitin (0.04 ± 0.002 U/mg)	GlcNAc and (GlcNAc)_2_ (α-chitin)	This study
Chib0434 from *Pseudoalteromonas flavipulchra* DSM 14401^T^	GH18	112.17	7.5	45	0	~80 min at 50 °C	α-chitin (0.01 ± 0.001 U/mg)	GlcNAc and (GlcNAc)_2_ (α-chitin)	This study
Chia4287 from *Pseudoalteromonas flavipulchra* DSM 14401^T^	GH18	50.86	7.0	50	0	<60 min at 50 °C	α-chitin (0.17 ± 0.005 U/mg)	GlcNAc and (GlcNAc)_2_ (α-chitin)	This study
CHI II from *Glaciozyma antarctica* PI12	GH18	39 and 50	4.0	15	-	<30 min at 30 °C	Colloidal chitin (-)	-	[26]
MmChi60 from *Moritella marina*	GH18	60.8	5.0	28	-	~5 h at 50 °C	Colloidal chitin (0.016 U/mg)	-	[29]
ChiA from *Pseudoalteromonas* sp. DL-6	GH18	113.5	8.0	20	-	~1 h at 40 °C	α-chitin (0.128 ± 0.001 U/mL)	(GlcNAc)_2_ (α-chitin)	[27]
ChiC from *Pseudoalteromonas* sp. DL-6	GH18	91	9.0	30	2	~1 h at 50 °C	α-chitin (4.8 ± 0.2 U/mg)	(GlcNAc)_2_ (colloidal chitin)	[37]
Chi23 from *Pseudoalteromonas aurantia* DSM6057	GH18	30.4	5.0	60	3	~40 min at 70 °C	Crystalline Chitin (0.1 ± 0.01 U/mg)	(GlcNAc)_2_ and GlcNAc)_3_ (α-chitin)	[18]
ChiB from *Pseudoalteromonas* sp. O-7	GH18	90.2	6.0	30	-	-	pNP-(GlcNAc)_2_ (30.8 U/mg)	-	[38]
ScChiC from *Streptomyces coelicolor* A3(2)	GH18	-	5	55	-	~1 h at 60 °C	(GlcNAc)_6_ (4120 ± 80 U/mg)	(GlcNAc)_2_ (crab shell chitin)	[19]
StmChiA from *Stenotrophomonas maltophilia*	GH18	70.5	5.0	40	-	>90% of initial activity at 30–50 °C (up to 1 h)	(GlcNAc)_6_ (-)	GlcNAc and (GlcNAc)_2_ (α-chitin)	[20]
StmChiB from *Stenotrophomonas maltophilia*	GH18	41.6	7.0	40	-	>90% of initial activity at 30–50 °C (up to 1 h)	(GlcNAc)_6_ (-)	-	[20]
PbChi67 from *Paenicibacillus barengoltzii* CAU904	-	67.9	3.5	60	-	43 min at 65 °C	α-chitin (0.3 ± 0.04 U/mg)	(GlcNAc)_2_, (GlcNAc)_3_ and(GlcNAc)_4_ (colloidal chitin)	[17]
A chitinase from *Bacillus* sp. R2	-	41.69	7.5	40	-	>30 min at 50 °C	Colloidal chitin (-)	-	[21]
A chitinase from *Citrobacter freundii* haritD11	-	64	8.0	35	-	~1 h at 60 °C	Colloidal chitin (140.55 U/mg)	-	[28]
A chitinase from *Micrococcus* sp. AG84	-	33	8.0	40	-	>1 h at 80 °C	Colloidal chitin (93.02 U/mg)	-	[22]
A chitinase from *Pseudoalteromonas* sp. DC14	-	65	9.0	40	10% (*w*/*v*)	>30 min at 60 °C	Colloidal chitin (5.6 U/mg)	-	[23]
A chitinase from *Streptomyces chilikensis* RC1830	-	10.5	7.0	60	-	-	Colloidal chitin (60.53 U/mg)	-	[24]
PtChi19 from *Pseudoalteromonas tunicata* CCUG 44952^T^	GH19	53.5	7.5	45	2	>40 min at 50 °C	Crystalline Chitin (16.4 mU/ mg)	-	[25]

- Not available.

## Data Availability

Proteomic data are available via ProteomeXchange with identifier PXD030600.

## Supplementary Materials

The following supporting information can be downloaded at: https://www.mdpi.com/article/10.3390/md20030165/s1, Figure S1: Multiple sequence alignments of Chib0431, Chib0434, Chia4287, and Chia2822 with known GH18 chitinases.; Figure S2: Phylogenetic analysis of Chib0889 with other GH19 chitinases.; Table S1: General information of 26 type strains of Pseudoalteromonas.; Table S2: Purification of the recombinant enzymes Chib0431, Chib0434, and Chia4287. References [39,74,75,76,77,78,79,80,81,82,83,84,85,86,87,88,89,90,91,92,93,94,95,96] are cited in the supplementary materials.
