# Supplementary material for: Identification and Characterization of Three Chitinases with Potential in Direct Conversion of Crystalline Chitin into N,N′-diacetylchitobiose
Source: Mar Drugs. 2022 Feb 24;20(3):165. doi: 10.3390/md20030165 (PMC8950537; doi:10.3390/md20030165)
Supplement: Supplementary file 1 [file marinedrugs-20-00165-s001.zip › marinedrugs-1574085-supplementary.pdf]

Identification and characterization of three chitinases with potentials in direct conversion of crystalline chitin into *N,N′*-diacetylchitobiose

Xue-Bing Ren[1], Yan-Ru Dang[1], Sha-Sha Liu[1], Ke-Xuan Huang[1], Qi-Long Qin[1,2];Xiu-Lan Chen[1,3], Yu-Zhong Zhang[1,2,3], Yan-Jun Wang[1,4]*;Ping-Yi Li[1]*

1. State Key Laboratory of Microbial Technology/Institute of Marine Science and Technology, Shandong University, Qingdao 266237, China

2. College of Marine Life Sciences, and Frontiers Science Center for Deep Ocean Multispheres and Earth System, Ocean University of China, Qingdao 266003, China

3. Laboratory for Marine Biology and Biotechnology, Pilot National Laboratory for Marine Science and Technology, Qingdao 266237, China

4. Institute of Wetland Agriculture and Ecology, Shandong Academy of Agricultural Sciences, Jinan 250100, China

* Correspondence: Author to whom correspondence should be addressed: Ping-Yi Li, lipingyipeace@sdu.edu.cn; Yan-Jun Wang, wang.yanjun1985@163.com.

Table S1 General information of 26 type strains of *Pseudoalteromonas*.

**Strain**	**Isolation source**	**Region**	**Reference**
P. agarivorans DSM 14585^T	Ascidians	Pacific Ocean	1
P. aliena DSM 16473^T	Seawater	Sea of Japan	2
P. arctica DSM 18437^T	Seawater	Arctic	3
P. aurantia DSM 6057^T	Seawater	Nice, France	4
P. carrageenovora DSM 6820^T	Seaweed	Japan	5
P. citrea DSM 8771^T	Seawater	Mediterranean Sea near Nice	6
P. espejiana DSM 9414^T	Seawater	the coast of Northern California, U.S.	7
P. flavipulchra DSM 14401^T	Seawater	Nice, France	8
P. issachenkonii DSM 15925^T	Brown alga	Pacific Ocean	9
P. lipolytica JCM 15903^T	Seawater	the Yangtze River estuary , China	10
P. luteoviolacea DSM 6061^T	Seawater	Nice, France	11
P. marina DSM 17587^T	Seawater	East Sea in Korea	12
P. mariniglutinosa DSM 15203^T	Diatom	Marseille Gulf	13
P. nigrifaciens DSM 8810^T	Mussels	Sea of Japan	14
P. paragorgicola DSM 26439^T	Gorgonian	Pacific Ocean	15
P. peptidolytica DSM 14001^T	Seawater	Sea of Japan	16
P. phenolica JCM 21460^T	Seawater	Ogasawara Island, Japan	17
P. piscicida JCM 20779^T	Red tide waters	southwest coast of Florida, U.S.	18
P. prydzensis DSM 14232^T	Sea ice	Antarctica	19
P. rubra DSM 6842^T	Seawater	Mediterranean, Nice, France	20
P. spongiae JCM 12884^T	Sponge	Hong Kong waters, China	21
P. tetraodonis DSM 9166^T	Puffer fish	Japan	22
P. translucida DSM 14402^T	Seawater	Sea of Japan	15
P. tunicata DSM 14096^T	Tunicate	western coast of Sweden	23
P. ulvae DSM 15557^T	Marine alga	Australia	24
P. undina DSM 6065^T	Seawater	the coast of Northern California, U.S.	7

A

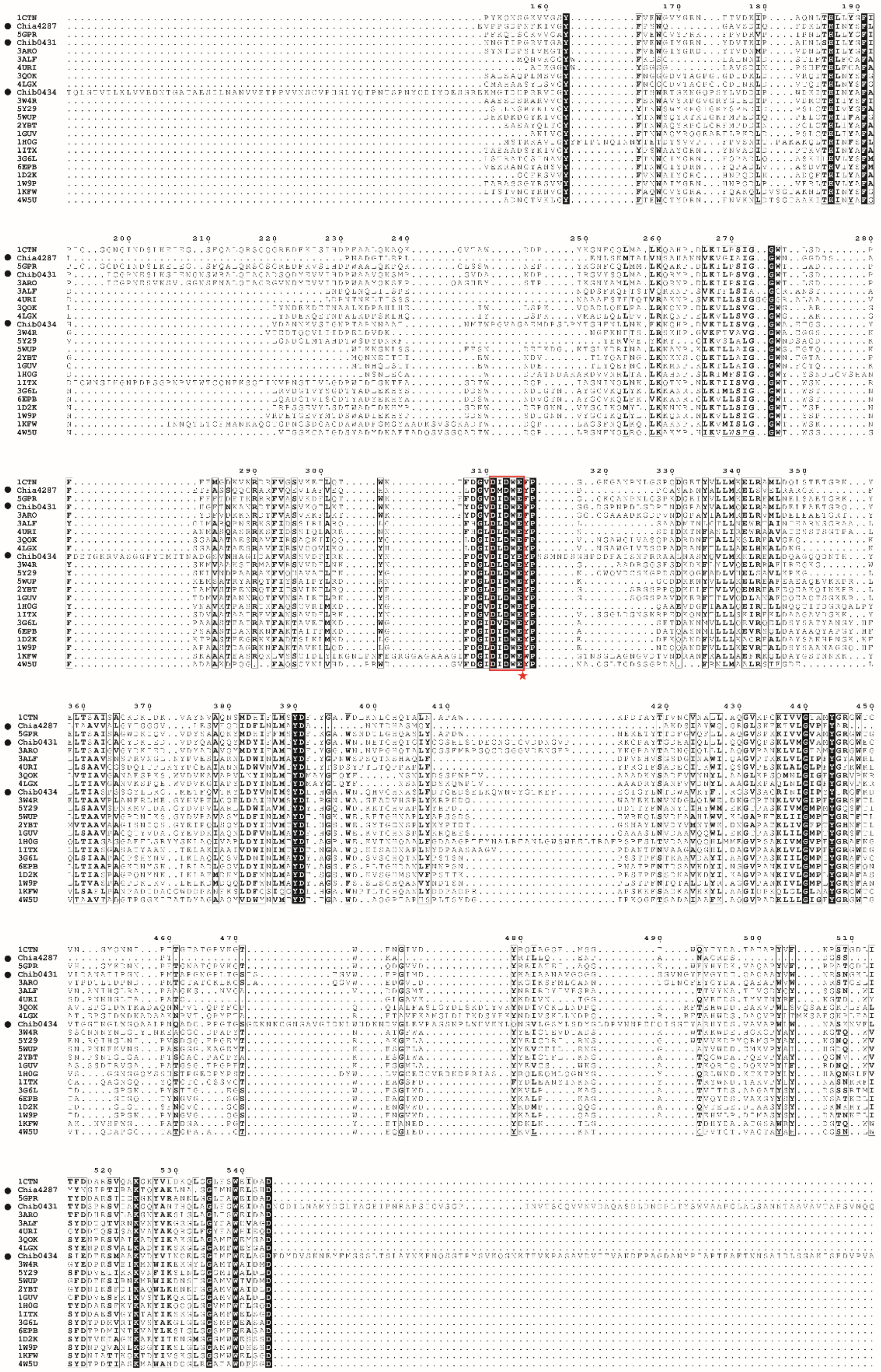

B

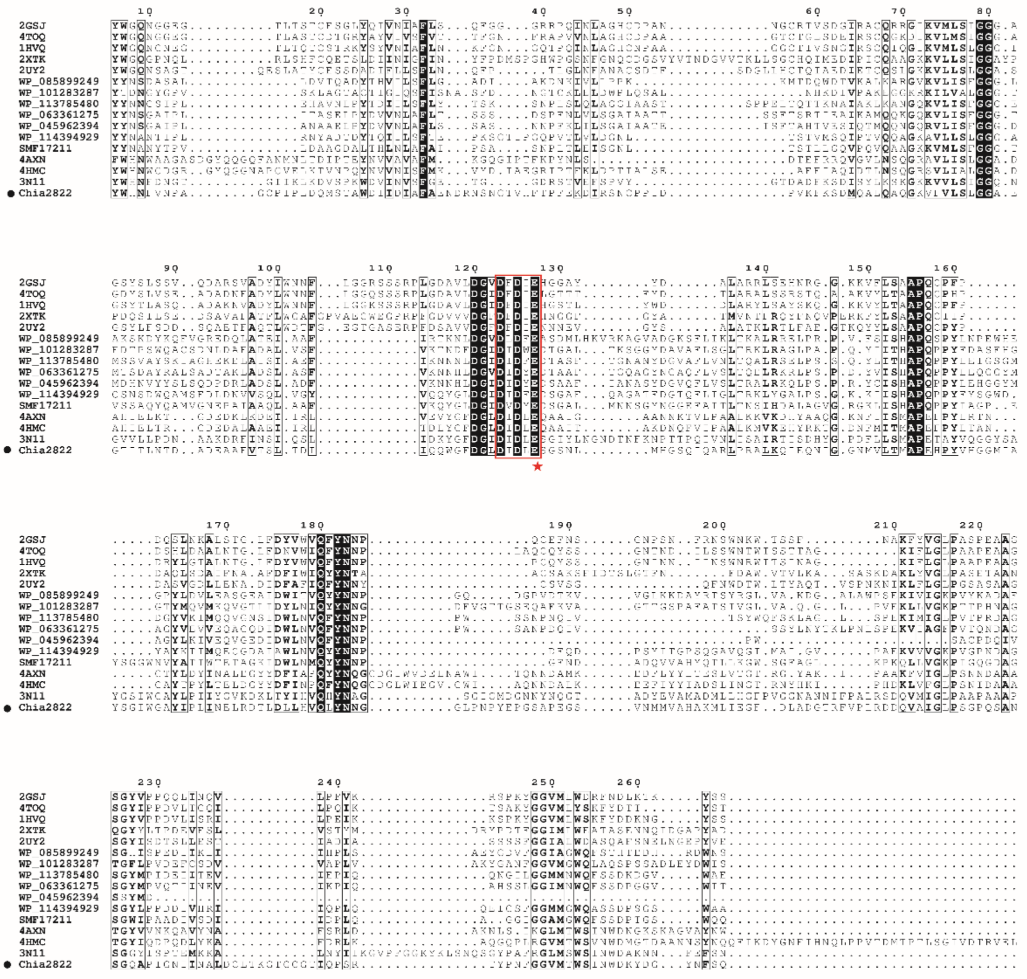

Figure S1. Multiple sequence alignments of Chib0431, Chib0434, Chia4287 and Chia2822 with known GH18 chitinases. The conserved catalytic motif (DxDxE) in GH18 chitinases is indicated by red boxes. (A) The alignments of Chib0431, Chib0434, Chia4287 with known GH18A subfamily chitinases. (B) The alignments of Chia2822 with known GH18B subfamily chitinases.

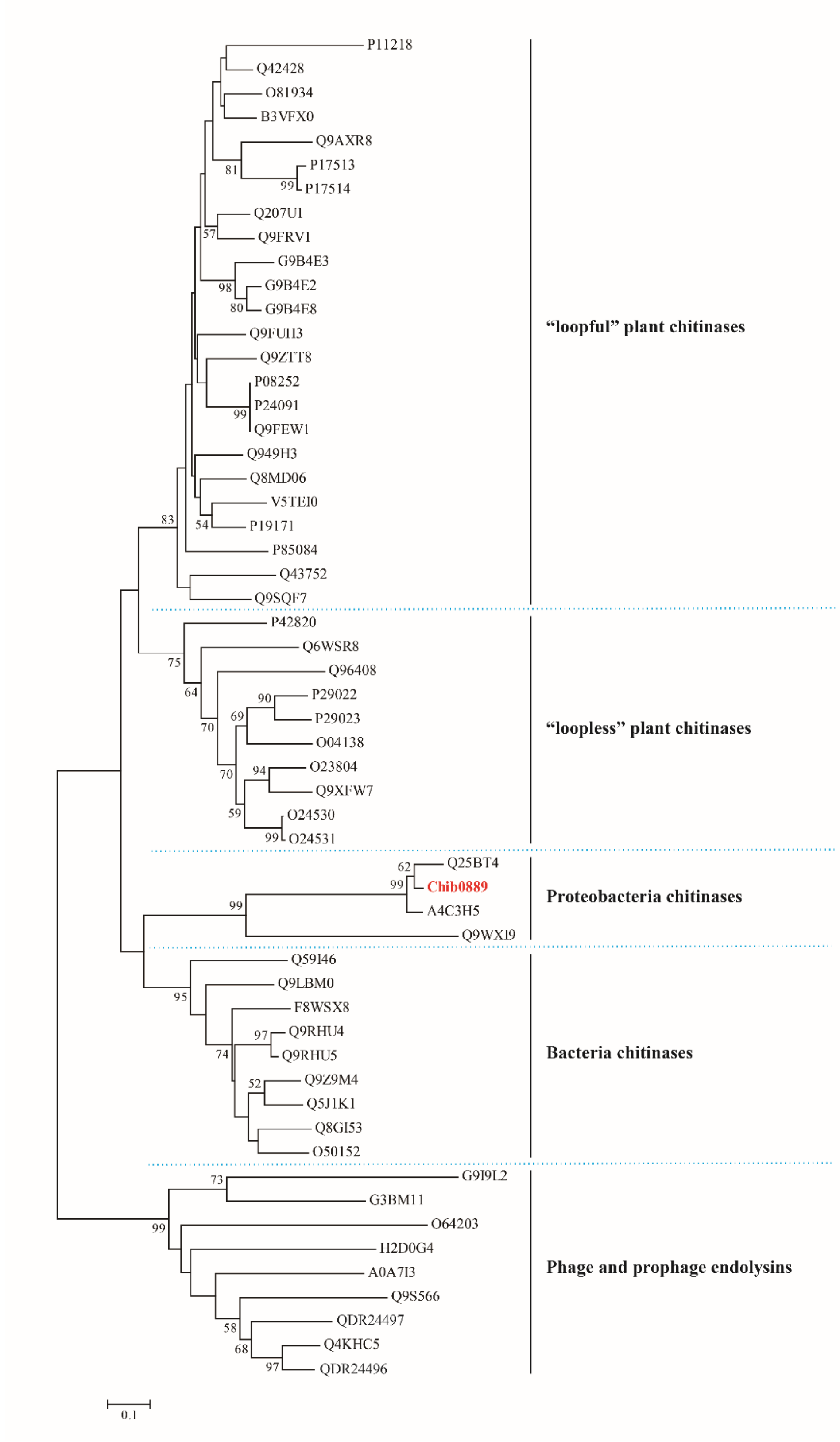

Figure S2. Phylogenetic analysis of Chib0889 with other GH19 chitinases. The phylogenetic tree was constructed by the Neighbor-Joining method. Bootstrap analysis of 1,000 replicates was conducted.

Table S2 Purification of the recombinant enzymes Chib0431, Chib0434 and Chia4287.

**Enzyme**	**Purification step**	**Total volume (mL)**	**Protein concentration (mg/mL)**	**Enzyme activity (U/mL)[a]**	**Total activity (U)**	**Specific activity (U/mg)**	**Purification fold[b]**	**Yield (%)[c]**
Chib0431	Cell extract	137	3.21	0.012	1.64	0.004	1	100
	Affinity chromatography	3.5	3.25	0.087	0.30	0.027	6.75	18.29
Chib0434	Cell extract	120	4.60	0.015	1.80	0.003	1	100
	Affinity chromatography	3.5	0.96	0.015	0.05	0.016	5.33	2.78
Chia4287	Cell extract	90	9.05	0.086	7.74	0.010	1	100
	Affinity chromatography	3.5	12.67	0.926	3.24	0.073	7.30	41.86

[a] Enzyme activity was measured with chitin powder as the substrate.

[b] (Ratio of the specific activity after purification tothat of the cell extract.

[c] Percentage of total activity recovered after purification compared with that of the cell extract.

Reference

1. Romanenko, L.A.; Zhukova, N.V.; Rohde, M.; Lysenko, A.M.; Mikhailov, V.V.; and Stackebrandt, E. *Pseudoalteromonas agarivorans* sp. nov., a novel marine agarolytic bacterium. *Int. J. Syst. Evol. Microbiol.* **2003**, 53, 125-131.
2. Ivanova, E.P.; Gorshkova, N.M.; Zhukova, N.V.; Lysenko, A.M.; Zelepuga, E.A.; Prokof'eva, N.G.; Mikhailov, V.V; Nicolau, D.V; Christen, R. Characterization of *Pseudoalteromonas distincta*-like sea-water isolates and description of *Pseudoalteromonas aliena* sp. nov. *Int. J. Syst. Evol. Microbiol.* **2004**, 54, 1431-1437.
3. Al Khudary, R.; Stosser, N.I.; Qoura, F.; Antranikian, G. *Pseudoalteromonas arctica* sp. nov., an aerobic, psychrotolerant,marine bacterium isolated from Spitzbergen. *Int. J. Syst. Evol. Microbiol.* **2008**, 58, 2018-2024.
4. Gauthier, M.; and Breittmayer, V.A. A new antibiotic-producing bacterium from seawater: *Alteromonas aurantia* sp. nov. *Int. J. Syst. Evol. Microbiol.* **1979**, 29, 366-372.
5. Akagawa-Matsushita, M.; Matsuo, M.; Koga, Y.; Yamasato, K. *Alteromonas atlantica* sp. nov. and *Alteromonas carrageenovora* sp. nov., bacteria that decompose algal polysaccharides. *Int. J. Syst. Evol. Microbiol.* **1993**, 42, 621-627.
6. Gauthier, M. *Alteromonas citrea*, a new Gram-negative, yellow-pigmented species from seawater. *Int. J. Syst. Evol. Microbiol.* **1977**, 27, 349-354.
7. Chan, K.; Baumann, L.; Garza, M.; Baumann, P. Two new species of *Alteromonas*: *Alteromonas espejiana* and *Alteromonas undina*. *Int. J. Syst. Evol. Microbiol.* **1978**, 28, 217-222.
8. Ivanova, E.P.; Shevchenko, L.S.; Sawabe, T.; Lysenko, A.M.; Svetashev, V.I.; Gorshkova, N.M.; et al. (). *Pseudoalteromonas maricaloris* sp. nov., isolated from an Australian sponge, and reclassification of [*Pseudoalteromonas aurantia*] NCIMB 2033 as *Pseudoalteromonas flavipulchra* sp. nov. *Int. J. Syst. Evol. Microbiol.* **2002**, 52, 263-271.
9. Ivanova, E.P.; Sawabe, T.; Alexeeva, Y.V.; Lysenko, A.M.; Gorshkova, N.M.; Hayashi, K.; Zukova, N.V.; Christen, R.; Mikhailov, V.V. *Pseudoalteromonas issachenkonii* sp. nov., a bacterium that degrades the thallus of the brown alga Fucus evanescens. *Int. J. Syst. Evol. Microbiol.* **2002**, 52, 229-234.
10. Xu, X.W.; Wu, Y.H.; Wang, C.S.; Gao, X.H.; Wang, X.G.; Wu, M. *Pseudoalteromonas lipolytica* sp. nov., isolated from the Yangtze River estuary. *Int. J. Syst. Evol. Microbiol.* **2009**, 60, 2176-2181.
11. Gauthier, M. Validation of the name *Alteromonas luteoviolacea*. *Int. J. Syst. Evol. Microbiol.* **1982**, 32(1), 82-86.
12. Yoon, J.H.; Kim, I.G.; Kang, K.H.; Oh, T.K.; Park, Y.H. *Alteromonas marina* sp. nov., isolated from sea water of the East Sea in Korea. *Int. J. Syst. Evol. Microbiol.* **2003**, 53, 1625-1630.
13. Romanenko, L.A.; Zhukova, N.V.; Lysenko, A.M.; Mikhailov, V.V.; Stackebrandt, E. Assignment of '*Alteromonas marinoglutinosa*'NCIMB 1770 to *Pseudoalteromonas mariniglutinosa* sp. nov., nom. rev., comb. nov. *Int. J. Syst. Evol. Microbiol.* **2003**, 53, 1105-1109.
14. Ivanova, E.P.; Kiprianova, E.A.; Mikhailov, V.V.; Levanova, G.F.; Garagulya, A.D.; Gorshkova, N.M.; Yumoto, N. Characterization and identification of marine *Alteromonas nigrifaciens* strains and emendation of the description. *Int. J. Syst. Evol. Microbiol.* **1996**, 46, 223-228.
15. Ivanova, E.P.; Sawabe, T.; Lysenko, A.M.; Gorshkova, N.M.; Hayashi, K.; Zhukova, N.V.; Nicolau, D.V.; Christen, R.; Mikhailov, V.V. *Pseudoalteromonas translucida* sp. nov. and *Pseudoalteromonas paragorgicola* sp. nov., and emended description of the genus. *Int. J. Syst. Evol. Microbiol.* **2002,** 52, 1759-1766.
16. Venkateswaran, K.; Dohmoto, N. *Pseudoalteromonas peptidolytica* sp. nov., a novel marine mussel-thread-degrading bacterium isolated from the Sea of Japan. *Int. J. Syst. Evol. Microbiol.* **2000**, 50, 565-574.
17. Isnansetyo, A.; Kamei, Y. *Pseudoalteromonas phenolica* sp. nov., a novel marine bacterium that produces phenolic anti-methicillin-resistant *Staphylococcus aureus* substances. *Int. J. Syst. Evol. Microbiol.* **2003**, 53, 583-588.
18. Hansen, A.; Weeks, O.; and Colwell, R. Taxonomy of *Pseudomonas piscicida* (Bein) Buck, Meyers, and Leifson. *J. Bacteriol.* **1965**, 89, 752-761.

19. Bowman, J.P. *Pseudoalteromonas prydzensis* sp. nov., a psychrotrophic, halotolerant bacterium from Antarctic sea ice. *Int. J. Syst. Evol. Microbiol.* **1998**, 48, 1037-1041.
20. Gauthier, M. *Alteromonas rubra* sp. nov., a new marine antibiotic-producing bacterium. *Int. J. Syst. Evol. Microbiol.* **1976**, 26, 459-466.
21. Lau, S.C.; Tsoi, M.M.; Li, X.; Dobretsov, S.; Plakhotnikova, Y.; Wong, P.-K.; Qian, P.Y. *Pseudoalteromonas spongiae* sp. nov., a novel member of the γ-Proteobacteria isolated from the sponge Mycale adhaerens in Hong Kong waters. *Int. J. Syst. Evol. Microbiol.* **2005**, 55, 1593-1596.
22. Ivanova, E.P.; Romanenko, L.A.; Matte, M.H.; Matte, G.R.; Lysenko, A.M.; Simidu, U.; Kita-Tsukamoto, K.; Sawabe, T., Vysotskii, M.V.; Frolova, G.M.; Mikhailov, V.; Christen, R.; Colwell, R,R. Retrieval of the species *Alteromonas tetraodonis* Simidu et al. 1990 as *Pseudoalteromonas tetraodonis* comb. nov. and emendation of description. *Int. J. Syst. Evol. Microbiol.* **2001**, 51, 1071-1078.
23. Holmström, C.; James, S.; Neilan; B.A., White, D.C.; Kjelleberg, S. *Pseudoalteromonas tunicata* sp. nov., a bacterium that produces antifouling agents. *Int. J. Syst. Evol. Microbiol.* **1998**, 48, 1205-1212.
24. Egan, S.; Holmström, C.; Kjelleberg, S. *Pseudoalteromonas ulvae* sp. nov., a bacterium with antifouling activities isolated from the surface of a marine alga. *Int. J. Syst. Evol. Microbiol.* **2001**, 51, 1499-1504.
